# Post-Surgery Circulating Tumor Cells and *AXL* Overexpression as New Poor Prognostic Biomarkers in Resected Lung Adenocarcinoma

**DOI:** 10.3390/cancers11111750

**Published:** 2019-11-07

**Authors:** Diego de Miguel-Pérez, Clara Isabel Bayarri-Lara, Francisco Gabriel Ortega, Alessandro Russo, María José Moyano Rodriguez, Maria Jesus Alvarez-Cubero, Elizabeth Maza Serrano, José Antonio Lorente, Christian Rolfo, María José Serrano

**Affiliations:** 1 Liquid Biopsy and Metastasis Research Group, GENYO, Centre for Genomics and Oncological Research, Pfizer/University of Granada/Andalusian Regional Government, PTS Granada, Avenida de la Ilustración 114, 18016 Granada, Spain; diego.miguel@genyo.es (D.d.M.-P.); franciscogabriel.ortega@ssib.es (F.G.O.); mjesusac@ugr.es (M.J.A.-C.); elisabeth.maza@juntadeandalucia.es (E.M.S.); jose.lorente@genyo.es (J.A.L.); 2 Laboratory of Genetic Identification, Legal Medicine and Toxicology Department, Faculty of Medicine, University of Granada. Av. de la Investigación, 11, 18071 Granada, Spain; 3 Marlene and Stewart Greenebaum Comprehensive Cancer Center, University of Maryland School of Medicine. 22 S. Greene Street, Baltimore, MD 21201, USA; alessandro-russo@alice.it; 4 Department of Thoracic Surgery, Virgen de las Nieves University Hospital, Av. de las Fuerzas Armadas, 2, 18014 Granada, Spain; clarai.bayarri.sspa@juntadeandalucia.es (C.I.B.-L.); maria.moyano.sspa@juntadeandalucia.es (M.J.M.R.); 5 Integral Oncology Division, San Cecilio Clinical University Hospital, Calle Dr. Oloriz 16, 18012 Granada, Spain

**Keywords:** NSCLC, *AXL*, circulating tumor cells, prognosis, EMT, adenocarcinoma, liquid biopsy, biomarkers

## Abstract

Background: The prognosis of early stage non-small cell lung cancer (NSCLC) is quite disappointing and the benefits of adjuvant therapy are relatively small. Thus, there is an urgent need to identify novel prognostic and predictive biomarkers. Lung adenocarcinoma has distinct clinical–pathological characteristics and novel therapeutic strategies are under active evaluation in the adjuvant setting. Here, we investigated the prognostic impact of circulating tumor cells (CTCs) and gene and miRNA tissue expression in resectable NSCLC. Patients and methods: We assessed the association between CTC subpopulations and the outcome of resected early stage lung adenocarcinoma (ADC) patients at three different time-points (CTC1-3) (before surgery, after one month, and after six months) in comparison to squamous cell carcinoma (SCC). Furthermore, gene and miRNA tissue expression, immunoprofiling, and epithelial-to-mesenchymal transition (EMT) markers were correlated with outcome. Results: ADC (*n* = 47) and SCC (*n* = 50) revealed different tissue expression profiles, resulting in the presence of different CTC subpopulations. In ADC, miR-155 correlated with *AXL* and *IL6R* expression, which were related to the presence of EMT CTC1 (*p* = 0.014 and *p* = 0.004). In the multivariate analysis, CTC2 was an independent prognostic factor for relapse-free survival, and CTC3 and *AXL* were independent prognostic for overall survival only in ADC. Neither the surgery nor the adjuvant treatment influenced the prognosis of these patients. Conclusions: Our study elucidate the prognostic impact of tissue *AXL* expression and the presence of CTCs after surgery in adenocarcinoma patients. Tissue *AXL* expression and CTC EMT activation could potentially represent biomarkers for the stratification of ADC patients that might benefit from new adjuvant therapies.

## 1. Background

Surgery is the mainstay of treatment for early stage non-small cell lung carcinoma (NSCLC) (stage I-IIIA), and the addition of platinum-based chemotherapy, either as adjuvant or neo-adjuvant therapy, has been associated with a modest but significant survival benefit [1]. Despite major breakthroughs in lung cancer biology and drug development in advanced/metastatic NSCLC, treatment of early stage disease has changed little over the last two decades [2]. The prognosis of resected NSCLC patients is highly heterogeneous and a proportion of stage IA patients, which are not candidate to adjuvant therapy, still recur after surgical resection. Furthermore, a high grade of heterogeneity exists in the outcome of stage IB-IIIA patients, even after aggressive post-surgical treatments that can include platinum-based chemotherapy with or without radiotherapy in selected cases. The biological mechanisms underlying these differences are not completely understood. 

Large whole exome sequencing studies revealed that squamous cell lung cancer (SCC) and lung adenocarcinoma (ADC) have distinct patterns of somatic genome alterations [3], as well as the clinical significance of intratumor heterogeneity, which could be therapeutically exploited [4]. In addition, ADC and SCC subtypes differ in prognosis and progression. In fact, SCC patients have lower survival rates after surgery [5]. These differences might be explained by the tumor mutation burden (TMB), as high nonsynonymous TMB was a favorable prognostic factor in resected NSCLC [6]. Nevertheless, despite the increasing knowledge about the biological behavior of these subtypes, the differences in the aggressiveness pattern between SCC and ADC are not clear yet.

Moreover, it is well known that circulating tumor cells (CTCs) contribute to tumor aggressiveness in advanced disease, disseminating through the blood to distant sites contributing to the process of metastatization of solid tumors, including NSCLC [7,8]. In contrast, only a few studies to date have evaluated the prognostic impact of CTCs in early stages of NSCLC [9,10,11]. However, the capacity of these CTCs to survive in different microenvironments and their success in colonizing new organs depends on the acquisition of genetic and phenotypic traits. This might be caused by the epithelial–mesenchymal transition (EMT) that, in neoplasias, consists of a cellular reprogramming that transforms epithelial cancer cells into mesenchymal or semi-mesenchymal cancer cells, allowing them to detach from the tumor and to disseminate into the blood. When the EMT is induced, the expression of transcriptions factors such as Zeb1 or Snail is increased. This, in turn, causes the loss of their classical polygonal morphology and the decrease in the expression of proteins such as E-cadherin. In exchange, they acquire a spindle-shaped morphology that is associated with an increase of mesenchymal proteins, such as vimentin and AXL. In addition, EMT has been associated with increased resistance to chemotherapy, targeted therapies, and immunotherapy [12,13].

As a consequence, heterogeneous subpopulations of CTCs can be found in the same patient [14]. In the current work, we analyze the presence of epithelial cells and mesenchymal cells and characterize their expression of epidermal growth factor receptor (EGFR), the overexpression of which has been associated with higher aggressiveness of NSCLC. On the other hand, microRNAs (miRNAs) are key regulators of gene expression and are therefore responsible for the phenotypical changes of tumor cells [15]. Specifically, miRNAs have pleiotropic actions and play key roles in several cellular processes, including cell proliferation, invasion, and EMT. Furthermore, miRNAs act as mediators of cell–cell communication and immune regulation. Aberrant miRNAs expression has been associated with several pathological conditions, including lung cancer, acting as biomarkers involved in the disease progression [16,17].

The aim of this study is to identify the specific correlation between gene and miRNA tissue expression with the release of CTC subpopulations and their role as prognostic biomarkers in adenocarcinoma and squamous cell carcinoma patients undergoing complete surgical resection. 

## 2. Patients and Methods

### Study Design and Patients

**Figure 1:**
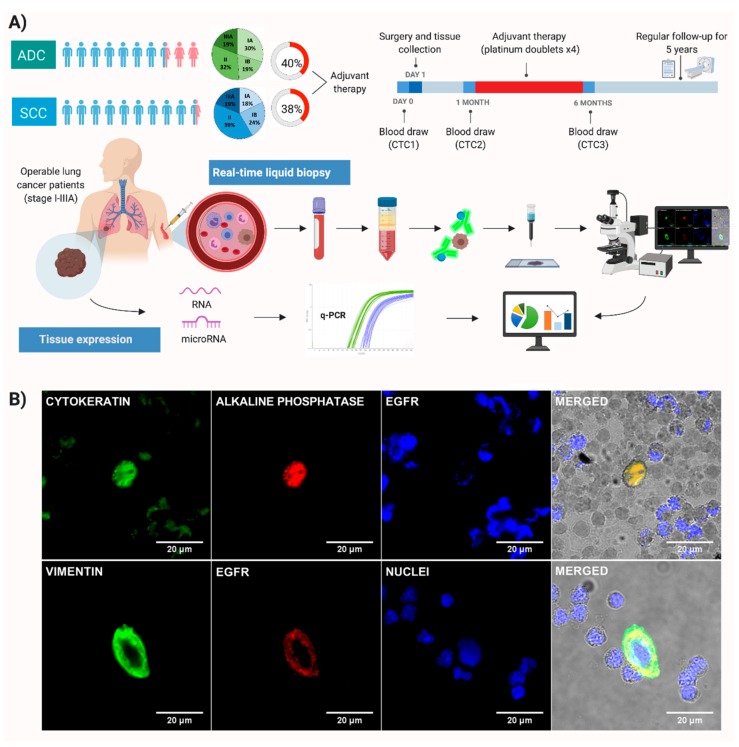
Study design and circulating tumor cell (CTC) characterization. (**A**) Patients enrolled and study design: this study included two cohorts, one of adenocarcinoma (ADC) and the other of squamous cell carcinoma (SCC) patients with similar distributions of gender, stage, and administered adjuvant treatment. Baseline blood sample (CTC1) was extracted before the surgery and tissue collection, one month after (CTC2), and six months later (CTC3) during adjuvant treatment in those susceptible cases (Credit: created with BioRender). (**B**) Immunofluorescence of CTC phenotype characterization in non-small cell lung cancer (NSCLC) patients: top row shows a cytokeratin-positive CTC (green and red staining) with epidermal growth factor receptor (EGFR) positive expression (blue). Bottom row represents an epithelial-to-mesenchymal transition (EMT) CTC with vimentin expression (green), EGFR positive expression (red), and DAPI—nuclei staining (blue). Images taken at 63× magnification in a Zeiss Epifluorescence Axio Imager A.1 microscope.

We conducted a prospective longitudinal cohort study in early stage NSCLC patients (I-IIIA) who underwent anatomical pulmonary resection and systematic lymph node dissection with curative intent at the University Hospital of Granada (Spain), between November 2012 and February 2015 (Supplementary Materials Text 1). Patients with concurrent or prior malignancy in the previous five years, with prior induction chemotherapy or radiotherapy, or who died within 30 days from the surgery were excluded. Pathological stage was defined according to the international tumor-node-metastasis (TNM) system seventh edition [18], which was the current version at the time of sample collection and histological diagnosis was posed using the World Health Organization classification. Indication for adjuvant treatment was established by the Local Tumor Committee according to the American Society of Clinical Oncology (ASCO) guidelines [19] and consisted of four cycles of platinum doublets. Clinical outcomes were evaluated in terms of relapse-free survival (RFS) and overall survival (OS). RFS was defined as the time from surgery until first recurrence (loco-regional or distant metastasis) or death due to any cause, while OS was the time from surgery until death due to any cause. Fresh tissue from the tumor pulmonary resection and 15 mL of peripheral blood were collected from each patient. Peripheral blood samples were extracted before surgery (2–16 h) (CTC1), one month later (CTC2), and six months later (CTC3) (during adjuvant treatment in those susceptible cases) (Figure 1A). Tissue and blood samples were analyzed at the GENyO Centre (Centre for Genomic and Oncological Research, Granada, Spain). In addition, this study included nine initially suspicious lung cancer patients that were confirmed as non-tumor in the resection and were used as tissue genetic expression controls and CTC negative controls. 

This study was approved by the Institutional Ethical Committee (Comité de Ética de la Investigación de Centro de Granada, CEI-GRANADA, Granada, Spain) on 26 July 2012 (ethic code: 27072012). Written informed consent was obtained from all cancer patients and healthy volunteers prior to inclusion in this study. Clinical studies were completed under good clinical and laboratory practice conditions in accordance with local standards.

## 3. Materials and Methods

### 3.1. Isolation and Characterization of CTCs

Peripheral blood samples (15 mL) were collected in EDTA tubes, stored at room temperature, and processed within 4 h after blood sampling. CTCs were isolated according to the previously established protocol by our group [11]. Briefly, blood samples were subjected to density gradient centrifugation and immunomagnetic isolation of epithelial cells with the Carcinoma Cell Enrichment and Detection Kit with MACS Technology (Miltenyi Biotec, Bergisch Gladbach, Germany), based on multicytokeratin antibody (CK3-11D5), which recognizes cytoplasmic cytokeratins 7, 8, 18, and 19. Each sample was spun down onto two slides in a cytocentrifuge (Hettich) and identified by immunocytochemical and chromogenic assays. Epithelial tumor cells were identified and counted based on their red staining. In positive slides, EGFR expression was analyzed by immunofluorescence [7]. On the other hand, in 54 baseline samples, MACS column elutes could be collected and subjected to a second immunomagnetic selection based on vimentin expression. Immunofluorescence techniques were applied to isolate and characterize EMT cells (EMT CTC1) (Figure 1B) (Supplementary Materials Text 2). 

### 3.2. Tissues Total RNA Extraction and cDNA Synthesis

Fresh tissues were stored at −80 °C in RNA later Tissue Protect Tubes (Qiagen, Hilden, Germany) until total RNA was extracted with TRIzolTM Reagent (Invitrogen, Waltham, MA, USA), according to manufacturer’s instructions. RNA concentration and purity were determined using NanoDrop 2000c Spectrophotometer (ThermoFisher Scientific, Waltham, MA, USA), 1 µg of total RNA was converted to complementary DNA (cDNA) according to the QuantiTect Reverse Transcription Kit (Qiagen) for subsequent RNA expression, and 10 ng of total RNA was converted to cDNA according to TaqManTM Advanced miRNA cDNA Synthesis Kit (ThermoFisher Scientific) for miRNAs analysis.

### 3.3. Quantitative (Real Time) Polymerase Chain Reaction for mRNA and miRNA Expression

Five genes were selected based on their reported association with tumor migration, aggressiveness, treatment resistance, immune response, and progression in NSCLC tumors (*AXL*, *IL6R*, *MET*, and *GAPDH*) [20,21,22,23]. In addition, five miRNAs (miR-21-5p, 222-3p, 155-5p, 24-3p, 30c-5p) were identified on their reported association with biological tumor characteristics and cisplatin/targeted therapies efficacy [16,24,25]. qRT-PCR primers were designed spanning exon–exon boundaries (Sigma-Aldrich, St. Louis, MO, USA), details in Supplementary Table S1.

In addition, B2M and miR-16 were selected as endogenous controls using Normfinder version 20 [26] and geNorm VBA applet for Microsoft Excel [27]. Gene expression was measured using the PerfeCta SYBR Green FastMix, Rox (Quanta Biosciences, Beverly, MA, USA) on a TaqMan 7900 HT Real-Time PCR system (Life Technologies, Carlsbad, CA, USA). Expression levels of miRNAs were measured using TaqManTM MicroRNA assays probes and TaqManTM Universal PCR Master Mix (ThermoFisher Scientific) in the same PCR System. Each test was run three times and included non-template controls (NTC). Expression levels are shown as 2^−ΔΔCt^ [28].

### 3.4. Statistical Analyses

CTCs were assessed as a continuous (number of CTCs) and as a binary variable (presence (CTCs ≥ 1)/absence). Cut-offs to distinguish between high and low levels of miRNA and gene expression were determined using the survival analysis of the Cutoff Finder web application [29]. The Kolmogórov–Smirnov test was applied and data were analyzed by Fisher’s exact test and non-parametric Kruskal–Wallis, Mann–Whitney U-test, and Spearman’s rank correlation tests. Univariate Kaplan–Meier (log-rank test) was used to analyzed CTC influence on RFS and OS, while Cox proportional hazard regression was applied for the multivariate analyses. Criterion of more than a 10% change in the variable coefficient estimate for the selection of variables to be included in the multivariate model was applied [30]. Statistical analyses were performed using IBM SPSS Statistics (version 22.0 for Windows, IBM Corp., Armonk, NY, USA) and graphs using GraphPad Prism (version 7.04 for Windows, GraphPad software, La Jolla, CA, USA). A *p*-value of <0.05 was considered statistically significant.

## 4. Results

A total of 97 consecutive NSCLC patients were enrolled in this study, including 47 ADC patients (median follow-up 30.5 months, range 3–50), and 50 SCC patients were used as a comparative NSCLC subpopulation (median follow-up 32 months, range 3–49). Clinical–pathological characteristics of both cohorts are summarized in Table 1. The two groups were well balanced, with no differences in terms of tumor stage and the proportion of patients undergoing adjuvant therapy (Figure 1A). As expected, the SCC group was associated with a lower percentage of women, larger tumor size, higher positron emission tomography—maximum standardized uptake values (PET SUVmax), and a higher percentage of pneumonectomy treatment compared with ADC.

### 4.1. Isolation and Characterization of CTCs

To evaluate the suitability of CTCs as prognostic factors in ADC and SCC patients, we isolated and characterized the different subtypes of CTCs before surgical resection (epithelial CTC1 and EMT CTC1) and during the follow-up (epithelial CTC2 and CTC3) (Figure 1B). No CTC was found in the control group of non-tumor patients. We compared the presence of CTCs at different time points in ADC and SCC, observing no significant differences between the two groups (Table 1). Moreover, clinical–pathological variables were compared to the presence of different phenotypes of CTCs at the different time points. In ADC patients, the presence of CTC2 was related to higher stages (*p* = 0.006), while the presence of CTC3 and EMT CTC1 was correlated with higher N stage (*p* = 0.017 and *p* = 0.007). On the contrary, no association was found between CTCs and clinical–pathological variables in SCC patients.

EGFR expression was analyzed in epithelial and EMT CTCs as observed in (Figure 1B). The frequency of patients with EGFR positive CTCs and the absolute number of these and other CTCs along the follow-up can be found in the Supplementary Table S2. All EMT CTCs had positive expression of EGFR. Moreover, in the 54 patients where both epithelial (CTC1) and EMT (EMT CTC1) were analyzed, 16 EMT and 130 epithelial CTCs were found. This resulted in a ratio of 1 EMT per 9.13 epithelial CTCs.

### 4.2. Genetic and miRNA Tissue Profiling and CTC Subpopulations

**Figure 2:**
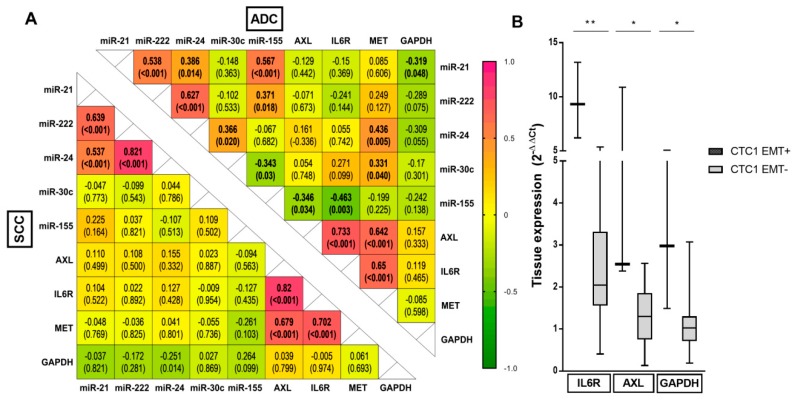
Genetic and miRNA tissue profiling and CTC correlation in ADC: (**A**): Heat-map correlation between selected miRNAs and genes in ADC (top-right corner) and in SCC (bottom-left corner). Data represent Spearman’s rho and (*p*-value). *p* < 0.05 in bold. Pink tones show positive correlations, yellow neutral, and green tones show negative correlations. (**B**): Blox-plot of gene tissue expression relationship with CTC phenotypes in ADC. Pattern fill colors represent the presence of CTC versus plain colors showing absence. Mann–Whitney U-test. * *p* < 0.05, ** *p* < 0.01.

We analyzed the specific tumor tissue miRNA and gene interactions involved in the pathogenesis of ADC versus SCC and the release of different subpopulations of CTCs in ADC patients in comparison to SCC. Interestingly, we observed specific correlations in ADC, as miR-155 inversely correlated with *IL6R* and *AXL* (*p* = 0.003 and *p* = 0.034, respectively) and, in contrast, *MET* expression positively correlated with miR-24 (*p* = 0.005) and miR-30c (*p* = 0.040) (Figure 2A). We found that higher expression of *IL6R*, *AXL*, and *GAPDH* correlated with the presence of EMT + CTC1 in these patients (*p* = 0.004, *p* = 0.014, and *p* = 0.021, respectively) (Figure 2B). On the other hand, no gene/miRNA and CTC correlation was observed in SCC patients.

### 4.3. Prognostic Markers of Relapse-Free Survival

A total of 23 (48.9%) ADC patients and 19 (38%) SCC patients relapsed during the follow-up (median follow-up 28 and 27.5 months, respectively). No significant differences were observed in the RFS between the two histological subtypes (*p* = 0.911). 

**Figure 3:**
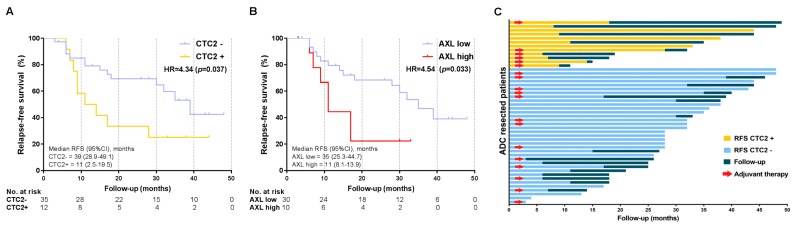
CTCs as relapse prognostic biomarkers and their dynamics in ADC: (**A**) Kaplan–Meier of the influence of CTC2 in the relapse-free survival (RFS). Patients with the presence of CTC2 had 4.34 times higher risk of developing relapse after the surgery versus those without them. Log-rank (Mantel–Cox) test was applied. (**B**) Kaplan–Meier of the influence of *AXL* tissue expression in the RFS. Patients with high *AXL* had 4.54 times higher risk of developing relapse after the surgery versus those with low expression. Log-rank (Mantel–Cox) test was applied. (**C**) Swimmer plot showing shorter RFS in patients with presence of CTCs after surgery (CTC2) (orange) versus patients with an absence of these cells (light blue). Follow-up after recurrence is represented in dark blue. Adjuvant therapy was administered after CTC2 extraction in those highlighted with red arrows, finding no significant differences according to the treatment between the two groups.

In the Kaplan–Meier analysis, the presence of CTC2 (Hazard Ratio (HR) = 4.34, *p* = 0.037) and high tissue levels of *AXL* (HR = 4.54, *p* = 0.033) were associated with worse RFS (Figure 3A,B) in ADC patients. *AXL* expression ranged from 2^−ΔΔCt^ = 0.088 to 10.895 and high levels were defined as 2^−ΔΔCt^ ≥ 1.796. The univariate Cox’s regression analysis was performed for each clinical–pathological and experimental variable. However, pneumonectomy (*p* = 0.047), N1 status (*p* = 0.06), adjuvant radiotherapy (*p* = 0.059), presence of CTC2 (*p* = 0.046), and high levels of *AXL* (*p* = 0.044) were the only variables associated with higher relapse risk in ADC patients (Supplementary Table S3). The EGFR CTCs, miRNAs, and other genes analyzed showed no statistical association. Neither the adjuvant chemotherapy nor the post-operative radiotherapy affected RFS. In the multivariate analysis, the presence of CTCs after surgery (CTC2) correlated with shorter RFS (Figure 3C) and was an independent prognostic factor for RFS in ADC patients (HR = 2.51, 95% CI = 1.07–5.87, *p* = 0.034) (Table 2).

On the contrary, no CTC subgroup or other experimental biomarkers were related to RFS in the SCC group (Supplementary Table S3). Tumor size and N status were the only independent prognostic factors for RFS in SCC patients (Table 2). 

### 4.4. Prognostic Markers of Overall Survival: Elucidating the Role of AXL

**Figure 4:**
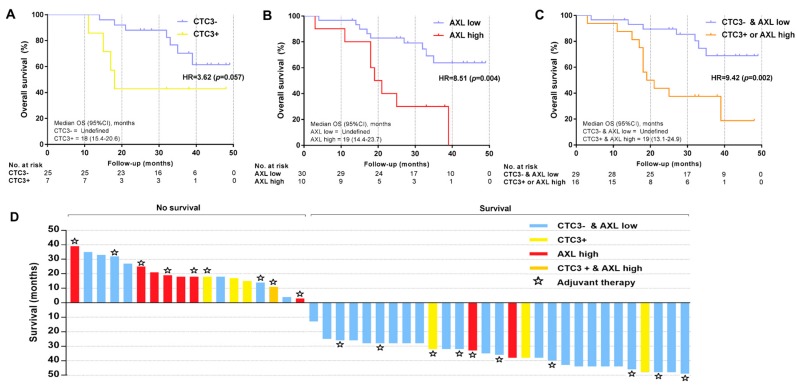
CTCs and tissue *AXL* as survival prognostic biomarkers in ADC: (**A**): Kaplan–Meier of the influence of CTC3 in the OS. Patients with presence of CTC3 had 3.62 times higher risk of exitus versus those without them. Log-rank (Mantel–Cox) test was applied. (**B**): Kaplan–Meier of the influence of *AXL* tissue expression in the OS. Patients with high *AXL* levels had an 8.51 times higher risk of exitus versus those with low levels. Log-rank (Mantel–Cox) test was applied. (**C**): Kaplan–Meier of the influence of CTC3 and *AXL* tissue expression in the OS. Patients with high *AXL* levels or presence of CTC3 had a 9.42 times higher risk of exitus versus patients with low *AXL* and negative CTC3. Log-rank (Mantel–Cox) test was applied. (**D**): Waterfall plot showing how presence of CTC3 (six months after surgery) (yellow), high *AXL* tissue expression (red), and both (orange) predicts OS in ADC patients, regardless adjuvant treatment administration (star).

A total of 18 (38.3%) ADC patients and 19 (38%) SCC patients died during the follow-up period (median follow-up 30.5 and 32 months, respectively), with no significant difference in OS between the two groups (*p* = 0.715). In the Kaplan–Meier analysis, the presence of CTC3 (HR = 3.62, *p* = 0.057) and high levels of tissue *AXL* (HR = 8.51, *p* = 0.004) were associated with a worse prognosis (Figure 4A,B) in ADC patients. When combined, CTC3+ and/or *AXL*-high patients had the shortest OS (HR = 9.42, *p* = 0.002) (Figure 4C). 

As previously reported for the RFS, each clinical–pathological and experimental variable was analyzed in the univariate analysis for OS. However, the presence of CTC2 was not an independent factor for OS. On the other hand, the presence of CTC3 (*p* = 0.072), high tissue *AXL* levels (*p* = 0.007), and high tissue *MET* levels (*p* = 0.002) were associated with shorter OS. *MET* expression ranged from 2^−ΔΔCt^ = 0.065 to 53.959 and high levels were defined as 2^−ΔΔCt^ ≥ 5.554. The EGFR CTCs, miRNAs, and other genes analyzed showed no statistical association. As previously observed with the RFS, neither adjuvant chemotherapy nor post-operative radiotherapy had an impact on the OS (Supplementary Table S4) (Figure 4D). The multivariate regression analysis revealed that the presence of CTC3 (HR = 10.8, 95% CI = 1.54–76.4, *p* = 0.017) and high *AXL* expression (HR = 15.7, 95% CI = 1.63–150.7, *p* = 0.017) were the only independent prognostic factors for OS in adenocarcinoma patients (Table 2). 

As reported for RFS, no CTC subgroup or experimental biomarkers were related to OS in the SCC group (Supplementary Table S4). The relapse and N status were the only independent prognostic factors for OS in SCC patients (Table 2).

## 5. Discussion

The clinical value of CTCs is constantly growing, as they can assist in precision medicine-based management of early resectable NSCLC patients by identifying those with an increased risk of recurrence. In this prospective study, we reported that the presence of heterogeneous CTC subpopulations is associated with tissue-specific genetic and miRNAs profiles in resected lung adenocarcinoma and how the role of the combination of both factors can identify patients with shorter RFS and OS. 

We observed that the different CTC subpopulations were correlated with gene and miRNAs interactions in the tissue. Interestingly, miR-155 expression was inversely correlated with *IL6R* and *AXL*, which were at the same time associated with the presence of EMT CTCs in adenocarcinoma. This concurs with previous studies, which reported that *IL-6R* and *AXL*, essential promoters of EMT in NSCLC through the STAT3 and the PI3K/AKT signaling pathways [31], are potential targets repressed by miR-155 [32]. EMT phenotype was also correlated with the expression of the Glyceraldehyde-3-phosphate dehydrogenase (*GAPDH*), which upregulates the EMT pathway [33] and is an adverse prognostic factor in resected NSCLC [21]. Hereby, our study describes, for the first time, how these genetic pathways could be involved in the dissemination of heterogeneous subpopulations of CTCs in ADC patients. Thus, a low miR-155/high *AXL–IL6R–GAPDH* tissue expression pathway might be involved in the release of CTCs with an EMT phenotype before surgery.

Furthermore, we studied the prognostic role of these CTC subpopulations and tissue biomarkers. According to our results, the presence of CTCs after surgery increased the risk of a shorter RFS more than 2-fold in ADC patients (HR = 2.51, 95% CI = 1.07–5.87, *p* = 0.034), regardless of adjuvant treatment. On the contrary, no effect on RFS was seen in the SCC subpopulation (HR = 1.18, 95% CI = 0.45–3.12, *p =* 0.733). Compared to advanced stages, there are only a few studies that have demonstrated the prognostic role of CTCs in resectable NSCLC [9,34,35], including two previous publications from our group [10,11]. 

In addition, we observed that the presence of CTCs at six months after surgery was a poor prognostic factor for OS in ADC, increasing the risk more than 10-fold (HR = 10.8, 95% CI = 1.54–76.4, *p* = 0.017). Interestingly, in these patients, high levels of *AXL* expression in tissue significantly decreased OS (more 15-fold; HR = 15.7, 95% CI = 1.63–150.7, *p* = 0.017). To the best of our knowledge, this is the first study that has reported the clinical impact of the presence of CTCs six months after surgery in the mortality of ADC patients, with no influence of the adjuvant treatment. In accordance with previous studies [23], we also confirmed that the overexpression of tissue *AXL* is a prognostic factor for survival in completely resected ADC patients.

Interestingly, miRNAs were not prognostic factors either in ADC or in SCC subpopulations. Moreover, we found that no CTC subpopulation or analyzed gene gave any prognostic value for RFS or OS in our cohort of SCC patients. We hypothesize that the differences in the prognostic value of the CTCs between both subtypes might be caused by the markers used in the isolation. Our study based the identification of epithelial and EMT CTCs according to the presence of cytokeratin and vimentin in both ADC and SCC patients. However, these subtypes differ in the molecular process of dissemination and pathogenesis. This suggests the need to identify new markers for the isolation of malignant subpopulations of CTCs in SCC, which could explain the differences in the aggressiveness between these two NSCLC subtypes.

On the other hand, we found that the presence of EMT CTCs at baseline status in ADC patients was associated with high N stage (*p* = 0.007), consistent with previous findings [36], relating the presence of these subpopulations to malignant progression. In addition, as reported in previous studies in early NSCLC [37], we found that EMT CTCs were associated with an increased risk of RFS (HR = 2.3) and OS (HR = 1.6), but with non-statistically significant association, perhaps due to the small sample size (EMT CTC1 detection performed in 22 ADC patients). Further studies including larger populations will be needed to confirm these results and address the prognostic significance of EMT CTCs in ADC patients. 

In the present study, we focused on *AXL* expression and EMT activation due to their important biological roles in lung cancer. Our study confirmed the poor prognostic survival role of *AXL* expression and showed a correlation between EMT activation on CTCs and malignant progression. Studies have proven how AXL increases the resistance to platinum-based chemotherapy as well as to EGFR targeted therapy in EMT cells [13]. In addition, *AXL* confers resistance to immunotherapy, promoting immune evasion by avoiding natural killer-cell activation and reducing innate cell-mediated anti-immune response. In lung adenocarcinoma, recent studies have confirmed that AXL inhibition decreased the expression of *PD-L1* and CXC chemokine receptor 6 (*CXCR6*), especially when *EGFR* was mutated [38]. More specifically, CTCs expressing PD-L1 present partial EMT phenotype in NSCLC and might represent a mechanism for immune escape [39]. The inhibition of AXL has been shown to enhance immune response following radiotherapy, therefore, targeting AXL could have a dual benefit, reducing EMT as well as activating the antitumor immune response [40]. All these data suggest that AXL inhibition could increase the sensitivity to platinum-based chemotherapy as well as anti-PD-1/PD-L1 therapies. Recently, immunotherapy has emerged as an effective treatment strategy in advanced/metastatic NSCLC and is rapidly moving in the neo/adjuvant setting with promising results [41]. Our study could provide the rationale for the inclusion of tissue *AXL* expression and CTC EMT activation as potential biomarkers of activity for these agents in early stage adenocarcinoma. In addition, these premises could open the door for combinations with AXL inhibitors in the adjuvant setting with the intent to increase the outcomes of resected NSCLC. 

## 6. Conclusions

Altogether, our study showed that the CTC presence one month after surgery was an independent prognostic factor for RFS, and the CTC presence six months after surgery and the tissue *AXL* expression were independent prognostic factors for OS in adenocarcinoma patients. Moreover, tissue *AXL* expression and CTC EMT activation could potentially represent biomarkers for the stratification of adenocarcinoma patients that might benefit from new adjuvant therapies.

## 7. Limitations of the Study

Although histological subtype populations were homogeneous, we consider that these results are limited by the low number of patients. There can be also inherent methodological limitations, due principally to the manual techniques used to analyze the presence of CTCs. Thus, to minimize bias and methodological variations, we have herein adopted rigorous standardized assay methods and the results were scored by two blinded independent well-trained clinical pathologists. Furthermore, additional studies with larger clinical sample cohort size from different centers would be of value to further validate our results.

## Figures and Tables

**Table 1:** Clinical–pathological and treatment-related characteristics of NSCLC patients by histological type.

Characteristics	NSCLC *n* = 97 (%)	ADC *n* = 47 (%)	SCC *n* = 50 (%)	*p*
**Gender**				
Men	84 (86.6%)	36 (76.6%)	48 (96%)	0.005
Women	13 (13.4%)	11 (23.4%)	2 (4%)	
**Age (years)**				
Mean ± SD	66.13 ± 8.65	65.49 ± 9.64	66.7 ± 7.6	
<70	58 (59.8%)	28 (59.6%)	30 (60%)	0.966
≥70	39 (40.2%)	19 (40.4%)	20 (40%)	
**Smoking habits**				
Never smoker	9 (9.3%)	7 (14.9%)	2 (4%)	0.168
Ex-smoker	62 (63.9%)	11 (23.4%)	15 (30%)	
Current smoker	26 (26.8%)	29 (61.7%)	33 (66%)	
**Stage**				
I	44 (45.4%)	23 (48.9%)	21 (42%)	0.700
II	25 (36.1%)	15 (31.9%)	20 (40%)	
III	18 (18.6%)	9 (19.1%)	9 (18%)	
**N status**				
N0	71 (73.2%)	34 (72.3%)	37 (74%)	0.909
N1	15 (15.5%)	8 (17%)	7 (14%)	
N2	11 (11.3%)	5 (10.6%)	6 (12%)	
**Tumor size (cm)**				
Mean ± SD	4.03 ± 2.13	3.52 ± 2.1	4.5 ± 2.1	
≤4 cm	54 (55.8%)	31 (57.4%)	23 (42.6%)	0.048
>4 cm	43 (44.3%)	16 (37.2%)	27 (62.8%)	
**PET (SUVmax)**				
Mean ± SD	11.05 ± 5.67	9.16 ± 5.06	12.74 ± 5.69	
≤9.4	47 (49.5%)	30 (66.7%)	17 (34%)	0.002
>9.4	48 (50.5%)	15 (33.3%)	33 (66%)	
**Surgical approach**				
Thoracotomy	57 (58.8%)	23 (48.9%)	34 (68%)	0.057
VATS	40 (41.2%)	24 (51.1%)	16 (32%)	
**Type of resection**				
Lobectomy	80 (82.5%)	43 (91.5%)	37 (74%)	0.024
Pneumonectomy	17 (17.5%)	4 (8.5%)	13 (26%)	
**Adjuvant chemotherapy**				
No	59 (60.8%)	28 (59.6%)	31 (62%)	0.807
Yes	38 (39.2%)	19 (40.4%)	19 (38%)	
**Adjuvant radiotherapy**				
No	91 (93.8%)	44 (93.6%)	47 (94%)	0.938
Yes	6 (6.2%)	3 (6.4%)	3 (6%)	
**CTC1**				
Mean (SD) Range	2.13 (± 8.7) 0–84	1.3 (± 2.6) 0–11	2.92 (± 11.9) 0–84	
Absence	57 (48.8%)	32 (68.1%)	25 (50%)	0.071
Presence	40 (41.2%)	15 (31.9%)	25 (50%)	
**CTC2**				
Mean (SD) Range	0.74 (± 1.6) 0–10	0.74 (± 1.9) 0–10	0.74 (± 1.2) 0–4	
Absence	70 (72.2%)	35 (74.5%)	35 (70%)	0.624
Presence	27 (27.8%)	12 (25.5%)	15 (30%)	
**CTC3**				
Mean (SD) Range	0.7 (± 1.8) 0–9	0.88 (± 2.1) 0–9	0.55 (± 2.8) 0–8	
Absence	57 (81.4%)	25 (78.1%)	32 (84.2%)	0.514
Presence	13 (18.6%)	7 (21.9%)	6 (15.8%)	
**CTC1 EMT**	**NSCLC *n* = 54 (%)**	**ADC *n* = 22 (%)**	**SCC *n* = 32 (%)**	
Mean (SD) Range	0.3 (± 0.44) 0–3	0.14 (± 0.4) 0–1	0.41 (± 0.8) 0–3	
Absence	43 (79.6%)	19 (86.4%)	24 (75%)	0.308
Presence	11 (20.4%)	3 (13.6%)	8 (25%)	

*p*: *p*-value of Fisher’s exact test between ADC and SCC; SD: Standard Deviation; TNM: Tumor Node Metastasis; N: node; PET (SUVmax): positron emission tomography—maximum standardized uptake values; VATS: video-assisted thoracic surgery.

**Table 2:** Final multivariate Cox proportional hazards regression model for RFS and OS in ADC and SCC.

ADC							
RFS	HR	95% CI	*p*	OS	HR	95% CI	*p*
Resection type				Relapse			
Pneumonectomy	4.23	1.13–15.8	0.032	Yes	15.0	1.04–216.2	0.047
Lobectomy	1.00			No	1.00		
CTC2				PET (SUVmax)			
Presence	2.51	1.07–5.87	0.034	>9.4	6.4	0.96–42.5	0.055
Absence	1.00			≤9.4	1.00		
				CTC3			
				Presence	10.8	1.54–76.4	0.017
				Absence	1.00		
				*AXL*			
				High	15.7	1.63–150.7	0.017
				Low	1.00		
**SCC**							
Size (cm)				Relapse			
>4cm	6.77	1.94–23.56	0.003	Yes	6.42	2.17–19.04	0.001
≤4cm	1.00			No	1.00		
N status				N status			
N0	1.00		0.192	N0	1.00		0.075
N1	2.72	0.92–7.98	0.070	N1	1.37	0.41–4.60	0.609
N2	1.26	0.28–5.84	0.764	N2	3.54	1.19–10.59	0.024

RFS: Relapse-free survival; OS: Overall survival; HR: hazard ratio; CI: confidence interval; *p*: *p*-value; PET (SUVmax): positron emission tomography—maximum standardized uptake values.

## Supplementary Materials

The following are available online at https://www.mdpi.com/2072-6694/11/11/1750/s1. Text 1: Clinical criteria; Text 2: Isolation and characterization of CTCs; Table S1: qRT-PCR primers sequence; Table S2: EGFR Expression in CTCs. Table 3: Univariate and multivariate Cox proportional hazards regression analysis for relapse-free survival; Table S4: Univariate and multivariate Cox proportional hazards regression analysis for overall survival.
