# Supplementary material for: Post-Surgery Circulating Tumor Cells and AXL Overexpression as New Poor Prognostic Biomarkers in Resected Lung Adenocarcinoma
Source: Cancers (Basel). 2019 Nov 7;11(11):1750. doi: 10.3390/cancers11111750 (PMC6896005; doi:10.3390/cancers11111750)
Supplement: Supplementary file 1 [file cancers-11-01750-s001.pdf]

Supplementary Materials: Post-surgery circulating tumor cells and *AXL* overexpression as new poor prognostic biomarkers in resected lung adenocarcinoma

Supplementary Text 1:

Clinical criteria:

Complete resection was defined as demonstrating cancer-free surgical margins, both grossly and histologically. Preoperative evaluation included history, physical examination, and laboratory and radiographic studies including PET CT scan. The follow-up schedule was the usually performed for resected NSCLC, consisting in a first clinical visit one month after surgery, then one every 3 months in the first year after resection and finally one every 6 months from the second to the fifth year. At least one chest CT scan was performed every 6 months and a PET-CT study per year. Local recurrence was defined as that occurring at the following sites: ipsilateral lung, bronchial stump or staple line, and regional lymph node (subcarinal, periesophageal, ipsilateral or contralateral mediastinum, supraclavicular, or hilar lymph nodes). Distant recurrence included metastases in the contralateral lung, liver, adrenal glands, brain, bone, or other locations. Patterns of recurrence were determined by clinical assessment, radiographical test and supplementary data from bronchoscopy, biopsy, and PET SUV.

Supplementary Text 2:

Isolation and characterization of CTCs

Paired epithelial samples slides were incubated with primary Goat Anti-EGFR (Santa Cruz biotech) and secondary Donkey anti-Goat IgG (H+L) Cross-Adsorbed Alexa Fluor 350 (Invitrogen). Finally, slides were mounted with SlowFade™ Antifade Kit (Invitrogen) and visualized under fluorescence microscope (Zeiss Epifluorescence Microscopy Zeiss Axio Imager A.1).

Baseline elutes resulted from the epithelial CTC isolation were subjected to a second immunomagnetic selection with primary Mouse Anti-Vimentin (FITC) (Santa Cruz biotech® sc-6260). They were incubated with Goat Anti-EGFR (Santa Cruz biotech® sc-31157) antibodies and subsequently with secondary Anti-FITC Microbeads (Miltenyi Biotec 130-048-701) and Donkey anti-Goat IgG (H+L) Cross-Adsorbed Alexa Fluor 555 (Invitrogen A-21432) antibodies. Finally, samples were eluted through a new magnetic column, spun down on a new slide and mounted with VECTASHIELD mounting medium with DAPI (Vector Labs) to be visualized under fluorescence microscope.

Table S1. qRT-PCR primers sequence.

Gene	Primer sequence
AXL	F: CAATGGGGACTACTACCGCC<br>R: GAAGGACCACACATCGCTCT
MET	F: CTGACTTGCTGAGAGGAGGC<br>R: GGTTTATCTTTCGGTGCCCAG
IL6R	F: TCAGTGTCACCTGGCAAGAC<br>R: GGAGGTCCTTGACCATCCAT
GAPDH	F: ATCACCATCTTCCAGGAGCGAGA<br>R: CATGGTTCACACCCATGACGAACA
β2M	F: TGCTGTCTCCATGTTTGATGTATCT<br>R: TCTCTGCTCCCCACCTCTAAGT

Table S2. EGFR Expression in CTCs.

	NSCLC		ADC		SCC	
**CTC (+) patients**	**EGFR+**	**EGFR-**	**EGFR+**	**EGFR-**	**EGFR+**	**EGFR-**
CTC1 (+) (n = 40)	25 (62.5%)	15 (37.5%)	12 (80%)	3 (20%)	13 (52%)	12 (48%)
EMT CTC1 (+) (n = 11)	11 (100%)	0 (0%)	3 (100%)	0 (0%)	8 (100%)	0 (0%)
CTC2 (+) (n = 27)	10 (37%)	17 (63%)	3 (25%)	9 (75%)	7 (46.7%)	8 (53.3%)
CTC3 (+) (n = 13)	6 (46.2%)	7 (53.8%)	3 (42.9%)	4 (57.1%)	3 (50%)	3 (50%)
**Number of CTCs**						
CTC1 (+) (n = 207)	117 (56.5%)	90 (43.5%)	31 (51.7%)	30 (48.3%)	86 (61.4%)	60 (38.6%)
EMT CTC1 (+) (n = 16)	16 (100%)	0 (%)	3 (100%)	0 (0%)	13 (100%)	0 (0%)
CTC2 (+) (n = 39)	18 (46.2%)	21 (53.8%)	5 (29.4%)	12 (70.6%)	13 (59.1%)	9 (40.9%)
CTC3 (+) (n = 22)	15 (68.2%)	7 (31.8%)	5 (55.6%)	4 (44.4%)	10 (76.9%)	3 (23.1%)

Table 3. Univariate and multivariate Cox proportional hazards regression analysis for relapse-free survival.

Characteristics	NSCLC						ADC						SCC					
	**Univariate**			**Multivariate**			**Univariate**			**Multivariate**			**Univariate**			**Multivariate**		
	**HR**	**95% CI**	***p***	**HR**	**95% CI**	***p***	**HR**	**95% CI**	***p***	**HR**	**95% CI**	***p***	**HR**	**95% CI**	***p***	**HR**	**95% CI**	***p***
**Age**	1.00	0.97-1.03	0.927				1.00	0.95-1.04	0.847				1.00	0.95-1.06	0.989			
**Gender**																		
Men	0.88	0.35-2.24	0.788				1.36	0.46-4.01	0.583				0.81	0.11-6.1	0.840			
Women	1.00						1.00						1.00					
**Histological type**																		
ADC	0.80	0.44-1.47	0.473															
SCC	1.00																	
**Stage**																		
I	1.00		0.004				1.00		0.046				1.00		0.087			
II	1.70	0.83-3.49	0.146				1.33	0.49-3.56	0.577				2.30	0.78-6.88	0.135			
III	3.75	1.71-8.24	0.001				3.53	1.28-9.78	0.015				4.04	1.56-14.14	0.029			
**Size (cm)**																		
>4cm	2.66	1.43-4.94	0.002	2.65	1.38-5.10	0.004	1.55	0.67-3.60	0.307				7.16	2.07-24.7	0.002	6.77	1.94-23.56	0.003
≤4cm	1.00			1.00			1.00						1.00			1.00		
**PET (SUVmax)**																		
>9.4	1.19	0.64-2.21	0.590				1.23	0.5-3.05	0.658				1.47	0.53-4.07	0.463			
≤9.4	1.00						1.00						1.00					
**N status**																		
N0	1.00						1.00		0.143				1.00		0.094	1.00		0.192
N1	2.85	1.36-5.95	0.006				2.76	0.96-7.94	0.06				3.22	1.16-9.27	0.031	2.72	0.92-7.98	0.070
N2	1.87	0.72-4.90	0.200				1.93	0.55-6.80	0.307				1.71	0.38-7.65	0.486	1.26	0.28-5.84	0.764
**Surgical approach**																		
Thoracotomy	1.70	0.88-3.27	0.113				1.47	0.64-3.36	0.361				2.68	0.78-9.21	0.117			
VATS	1.00						1.00						1.00					
**Resection type**																		
Pneumonectomy	1.56	0.75-3.27	0.236				3.72	1.02-13.63	0.047	4.23	1.13-15.8	0.032	1.42	0.54-3.73	0.482			
Lobectomy	1.00						1.00			1.00			1.00					
**Adjuvant chemotherapy**																		
Yes	1.69	0.92-3.10	0.088				1.73	0.77-3.93	0.188				1.56	0.63-3.84	0.335			
No	1.00						1.00						1.00					
**Adjuvant radiotherapy**																		
Yes	2.92	1.14-7.46	0.025				3.29	0.96-11.33	0.059				2.58	0.59-11.22	0.206			
No	1.00						1.00						1.00					
**CTC1 (number)**	0.96	0.86-1.06	0.416				0.96	0.82-1.11	0.553				0.94	0.76-1.16	0.566			
**CTC1**																		

Presence	0.98	0.53-1.81	0.953				1.44	0.62-3.35	0.395				0.74	0.3-1.82	0.511
Absence	1.00						1.00						1.00		
**EGFR+ CTC1**															
Presence	0.89	0.45-1.78	0.744				1.25	0.51-3.04	0.625				0.66	0.22-1.98	0.454
Absence	1.00						1.00						1.00		
**CTC2 (number)**	1.12	0.97-1.29	0.120				1.13	0.97-1.32	0.113				1.13	0.79-1.62	0.513
**CTC2**															
Presence	1.61	0.86-3.03	0.139				2.40	1.01-5.49	0.046	2.51	1.07-5.87	0.034	1.18	0.45-3.12	0.733
Absence	1.00						1.00			1.00			1.00		
**EGFR+ CTC2**															
Presence	0.57	0.18-1.84	0.348				1.98	0.46-8.56	0.361				0.27	0.03-1.99	0.197
Absence	1.00						1.00						1.00		
**CTC3 (number)**	1.04	0.87-1.24	0.657				1.02	0.79-1.32	0.885				1.09	0.86-1.38	0.497
**CTC3**															
Presence	1.75	0.78-3.91	0.174				1.60	0.51-5.0	0.418				2.02	0.64-6.34	0.231
Absence	1.00						1.00						1.00		
**EGFR+ CTC3**															
Presence	1.44	0.36-5.79	0.606				1.86	0.26-13.51	0.540				1.32	0.18-9.53	0.786
Absence	1.00						1.00						1.00		
**EMT CTC1**															
Presence	0.51	0.15-1.74	0.285				2.29	0.46-11.35	0.312				0.19	0.24-1.45	0.108
Absence	1.00						1.00						1.00		
AXL															
High	0.74	0.39-1.39	0.346				2.65	1.03-6.81	0.044				0.52	0.17-1.57	0.243
Low	1.00						1.00						1.00		
IL6R															
High	1.35	0.68-2.67	0.398				1.70	0.67-4.32	0.264				2.48	0.97-6.30	0.057
Low	1.00						1.00						1.00		
MET															
High	0.65	0.34-1.25	0.200				1.78	0.73-4.34	0.204				0.50	0.16-1.51	0.216
Low	1.00						1.00						1.00		
GAPDH															
High	3.34	1.30-8.55	0.012	2.78	1.07-7.18	0.035	8.31	1.11-62.03	0.039				0.21	0.03-1.57	0.129
Low	1.00			1.00			1.00						1.00		
**miR-21**															
High	1.24	0.65-2.38	0.520				0.40	0.91-1.74	0.220				0.21	0.03-1.57	0.129
Low	1.00						1.00						1.00		
**miR-222**															
High	1.54	0.79-2.98	0.198				2.07	0.80-5.38	0.135				3.10	1.05-9.20	0.041
Low	1.00						1.00						1.00		
**miR-24**															
High	1.63	0.83-3.23	0.158				1.65	0.69-3.94	0.260				2.40	0.86-6.66	0.093
Low	1.00						1.00						1.00		

**miR-30c**									
High	0.53	0.27-1.02	0.056	1.43	0.59-3.46	0.428	0.29	0.10-0.81	0.018
Low	1.00			1.00			1.00		
**miR155**									
High	0.68	0.32-1.45	0.324	1.68	0.70-4.00	0.243	0.58	0.21-1.60	0.291
Low	1.00			1.00			1.00		

HR: hazard ratio; CI: confidence interval; *p*: *p*-value; N: node; VATS: video-assisted thoracic surgery.

Table S4. Univariate and multivariate Cox proportional hazards regression analysis for overall survival.

	NSCLC						ADC						SCC					
	Univariate			Multivariate			Univariate			Multivariate			Univariate			Multivariate		
**Characteristics**	**HR**	**95% CI**	***p***	**HR**	**95% CI**	***p***	**HR**	**95% CI**	***p***	**HR**	**95% CI**	***p***	**HR**	**95% CI**	***p***	**HR**	**95% CI**	***p***
**Age**	1.02	0.98-1.06	0.449				1.03	0.97-1.09	0.385				1.01	0.95-1.06	0.872			
**Gender**																		
Men	1.81	0.55-5.89	0.327				1.36	0.39-4.74	0.630				21.81	0.0-1.9x10^5	0.506			
Women	1.00						1.00						1.00					
**Histological type**																		
ADC	1.05	0.55-2.00	0.882															
SCC	1.00																	
**Relapse**																		
Yes	6.10	2.77-13.41	<0.001	3.70	1.61-8.54	0.002	5.07	1.62-19.44	0.007	15.0	1.04-216.2	0.047	6.91	2.43-19.66	<0.001	6.42	2.17-19.04	0.001
No	1.00			1.00			1.00			1.00			1.00			1.00		
**Stage**																		
I	1.00						1.00		0.036				1.00		0.028			
II	1.79	0.80-4.01	0.154				1.81	0.58-5.63	0.303				1.77	0.56-5.58	0.331			
III	4.47	1.99-10.03	<0.001				4.46	1.42-13.97	0.010				4.60	1.45-15.58	0.01			
**Size (cm)**																		
>4cm	3.23	1.64-6.37	0.001	3.40	1.56-7.41	0.002	3.54	1.34-9.02	0.008				3.19	1.14-8.90	0.027			
≤4cm	1.00			1.00			1.00						1.00					
**PET (SUVmax)**																		
>9.4	1.57	0.80-3.10	0.189				2.56	0.96-6.84	0.061	6.4	0.96-42.5	0.055	1.05	0.4-2.75	0.928			
≤9.4	1.00						1.00			1.00			1.00					
**N status**																		
N0	1.00		0.001	1.00		0.02	1.00		0.007				1.00		0.019	1.00		0.075
N1	3.75	1.73-8.12	0.001	2.46	1.05-5.76	0.038	5.83	1.93-17.55	0.002				2.83	0.88-9.09	0.081	1.37	0.41-4.60	0.609
N2	2.96	1.25-7.02	0.014	3.30	1.23-7.50	0.016	1.63	0.34-7.49	0.528				4.28	1.46-12.58	0.008	3.54	1.19-10.59	0.024
**Surgical approach**																		
Thoracotomy	1.56	0.78-3.11	0.205				1.94	0.75-5	0.172				1.25	0.45-3.46	0.674			
VATS	1.00						1.00						1.00					
**Resection type**																		
Pneumonectomy	2.49	1.23-5.05	0.011				5.67	1.51-21.33	0.010				2.20	0.88-5.46	0.091			
Lobectomy	1.00						1.00						1.00					
**Adjuvant chemotherapy**																		
Yes	1.73	0.91-3.29	0.098				1.58	0.63-3.98	0.334				1.87	1.75-4.53	0.186			
No	1.00						1.00						1.00					

**Adjuvant radiotherapy**															
Yes	2.00	0.70-5.70	0.194				2.07	0.46-9.2	0.341				1.93	0.44-8.41	0.383
No	1.00						1.00						1.00		
**CTC1 (number)**	0.94	0.82-1.09	0.421				0.94	0.76-1.17	0.577				0.94	0.78-1.14	0.527
**CTC1**															
Presence	0.95	0.49-1.83	0.870				1.45	0.56-3.74	0.448				0.65	0.26-1.61	0.347
Absence	1.00						1.00						1.00		
**EGFR+ CTC1**															
Presence	1.02	0.49-2.10	0.968				1.56	0.59-4.18	0.372				0.67	0.22-2.02	0.476
Absence	1.00						1.00						1.00		
**CTC2 (number)**	1.12	0.97-1.31	0.132				1.14	0.96-1.35	0.134				1.10	0.76-1.59	0.617
**CTC2**															
Presence	1.47	0.75-2.89	0.264				1.72	0.66-4.45	0.264				1.22	0.46-3.22	0.685
Absence	1.00						1.00						1.00		
**EGFR+ CTC2**															
Presence	0.42	0.10-1.76	0.240				0.77	0.10-5.83	0.804				0.28	0.04-2.11	0.217
Absence	1.00						1.00						1.00		
**CTC3 (number)**	0.95	0.76-1.19	0.643				0.99	0.76-1.29	0.943				0.90	0.60-1.34	0.595
**CTC3**															
Presence	1.61	0.64-4.02	0.312				3.12	0.91-10.76	0.072	10.8	1.54-76.4	0.017	0.84	0.19-3.73	0.816
Absence	1.00						1.00			1.00			1.00		
**EGFR+ CTC3**															
Presence	1.11	0.22-5.54	0.897				1.41	0.20-10.19	0.731				0.71	0.04-11.79	0.809
Absence	1.00						1.00						1.00		
**EMT CTC1**															
Presence	0.55	1.60-1.91	0.347				1.65	0.29-9.21	0.571				0.21	0.03-1.65	0.212
Absence	1.00						1.00						1.00		
AXL															
High	2.13	0.82-5.52	0.121				3.80	1.44-9.99	0.007	15.7	1.63-150.7	0.017	0.47	0.17-1.32	0.150
Low	1.00						1.00			1.00			1.00		
IL6R															
High	1.31	0.64-2.69	0.458				2.02	0.76-5.32	0.157				0.42	0.00-40.56	0.366
Low	1.00						1.00						1.00		
MET															
High	1.81	0.91-3.62	0.091	3.09	1.47-6.52	0.003	3.03	1.17-7.87	0.023				1.86	0.74-4.72	0.189
Low	1.00			1.00			1.00						1.00		
GAPDH															
High	2.33	1.12-4.85	0.024				32.41	0.32-3311.44	0.141				2.23	0.88-5.67	0.092
Low	1.00						1.00						1.00		
**miR-21**															
High	0.30	0.07-1.25	0.099				0.24	0.03-1.84	0.170				1.48	0.55-3.99	0.437
Low	1.00						1.00						1.00		

**miR-222**									
High	1.42	0.72-2.82	0.315	2.73	0.89-8.39	0.079	0.55	0.20-1.53	0.253
Low	1.00			1.00			1.00		
**miR-24**									
High	1.88	0.91-3.92	0.090	2.91	0.95-8.98	0.063	0.48	0.17-1.32	0.154
Low	1.00			1.00			1.00		
**miR-30c**									
High	0.21	0.03-1.56	0.129	1.33	0.51-3.47	0.555	0.39	0.13-1.21	0.102
Low	1.00			1.00			1.00		
**miR155**									
High	0.39	0.12-1.29	0.123	0.51	0.12-2.27	0.380	0.27	0.04-2.08	0.209
Low	1.00			1.00			1.00		

HR: hazard ratio; CI: confidence interval; *p*: *p*-value; N: node; VATS: video-assisted thoracic surgery.
